# Physicians’ perspective regarding direct to consumer marketing of nutraceuticals products

**DOI:** 10.12669/pjms.343.15120

**Published:** 2018

**Authors:** Kamran Zaman, Kiran Asim, Nusrat Shah, Syed Jamshed Ahmed

**Affiliations:** 1 Kamran Ali Zaman, Pharm-D, MBA. Marketing Manager and CRA, PharmEvo (Pvt.) Ltd, Karachi, Pakistan; 2 Dr. Kiran Asim, MBBS. Resident Medical Officer, Allied Hospital, Faisalabad, Pakistan; 3 Prof. Nusrat Shah, Professor of Gynecology and Obstetrics, Dow University of Health Sciences, Karachi, Pakistan; 4 Syed Jamshed Ahmed, B-Pharm, MBA, PhD Scholar Deputy Chief Executive Officer, PharmEvo (Pvt.) Ltd., Director Pharmacy Services, Alkhidmat Hospital, Karachi, Pakistan

**Keywords:** Nutraceuticals, Direct to consumer advertisement, E-marketing, Medications

## Abstract

**Objectives:**

Recently pharmaceutical marketers have expanded their audience by directly to the consumers advertising (DTCA) which is almost always limited to non-prescription drugs. DTCA has substantial effects on patient behavior and physician prescription. The aim of this study was to assess the perspectives of physicians regarding the rapidly proliferating trend of direct to consumer marketing of nutraceutical drugs (ND).

**Methods:**

It was a cross-sectional study from June 2016 to December 2016 which included 325 physicians from various cities of Pakistan who completed a structured questionnaire after providing informed consent. Questions were asked to assess physicians’ perception of the increasing trend of Direct to Consumer advertisement of nutraceuticals and its influence on their practice. The data was analyzed using SPSS (SPSS Inc., Chicago, Illinois, USA).

**Results:**

There were 182 (56%) male and 143 (44%) female physicians in the study. Most of them were general practitioners (48%), spent an average weekly time of 1-2 hours gaining medical knowledge (56%), and most of them (52%) utilized internet as their source. Most physicians (88%, n=286) experienced knowledgeable patients who inquired about their diseases and treatment plans. Most of the physicians believed that e-detailing (72%, n=234) and DTCA (68%, n=221) of nutraceuticals helps practitioners in shaping a more effective treatment plan. Almost (62%, n=201) physicians prescribed medications their patients requested them to.

**Conclusion:**

Physician perception of DTCA and e-detailing of nutraceuticals seems to be promising. However, physicians must be more prepared to deal with knowledgeable patients and put in maximum efforts to counsel them in such a manner that the prescription doesn’t contain “request specific” drugs but only the ones that are most beneficial for the patients.

## INTRODUCTION

Globally, the shift in trend from prescription drug to over-the-counter and natural substances for prevention and treatment of diseases has been observed. Global demand for herbal and non-herbal extracts is increasing continuously. Until recently, pharmaceutical marketing, drug detailing, and product advertising was only limited to the physicians who were actually the customers.^1^ However, recently pharmaceutical marketers have expanded their audience by advertising directly to the consumers. It is not legal for pharmaceuticals to directly market Prescription Drugs (PD), hence Direct To Consumer Advertising (DTCA) is almost always limited to Nutraceutical Drugs (ND) and usually include dietary supplements, nutritionally fortified or engineered foods, beverages and food ingredients that promote health and reduce disease risk.^2^

Ever since the idea of DTCA gained popularity, researchers started to pay more heed to it. Researchers have highlighted how DTCA results in irrelevant request of a certain drug brand, misuse of medicines and ultimately higher treatment expense.^3^ However, other researchers have argued that DTCA results in increased knowledge of the disease, its concerned ND, and eventually motivates people to seek proper medical treatment.^4^ It was seen that patients find familiar and recommended drugs brands more comfortable and reliable and try to extract additional information from their physicians about the advertised drug brand.^5^ DCTA also has a dual impact on the healthcare seeking behavior of the individuals.^6^

As far as the attitude of physicians towards DCTA of ND is concerned, some researchers have found that DTCA results in irrational prescribing of drugs by the physicians, but it also helps spreading disease knowledge to the hard to reach parts of popsulation.^7^ Studies have proved the role of drug advertising in physician’s preferred choice of prescription drugs.^8^ Patient-requested prescription as a result of DCTA was commonly seen among women patients and people with high cognition. These patients were also more satisfied with their physician.^9^ As a result of journal advertising, detailing and sample distribution, the demand and popularity of a certain drug brand increase which helps in generating more sales.^10^

Developed countries are investing more in nutrient market than in their drug market. 11 However, there is still limited data from developing countries. 12 Since nutraceuticals have a more substantial role in disease prevention and delaying complications of chronic diseases; 13 specialists in public health should look into this mode of disease management carefully and plan strategies for mass awareness about nutraceuticals and their benefits.

Aim of this research was to study the perspectives of physicians regarding the rapidly proliferating trend of direct to consumer marketing of nutraceuticals. Since the objective of pharmaceutical companies behind DTCA is to influence physicians’ prescriptions, the rationale of this study is to justify additional investment of pharmaceutical companies in this sector. The second aim of this study was to contribute comparable evidences from developing countries like Pakistan, where regulation and implementation of drug laws is a major challenge.

## METHODS

It was a cross-sectional study that included 325 physicians from various cities of Pakistan. Practicing physicians with clinical practice of minimum five years from internal medicine, obstetrics and gynecology, and general practitioners were included in this study after taking informed consent. Four hundred physicians were approached for the study, however, three hundred and twenty five completed the study questionnaire and were included (response rate 81.25%). The questionnaire included their sociodemographic details and questions about their perception of the increasing trend of Direct to Consumer advertisement of nutraceuticals and its influence on their practice. The data was collected by two research assistants who were trained by first author. The data was analyzed using SPSS 23 (SPSS Inc, Chicago, Illinois, USA).

## RESULTS

Of the 325 physicians who completed the questionnaire, 182 (56%) were males and 143 (44%) were females. Most of them were general practitioners (48%), spent an average weekly time of one to two hours gaining medical knowledge (56%), and most of them (52%) utilized internet as their source of gaining medical knowledge (Table-I).

**Table-I:** Characteristics of the physicians participating in the study (n=325).

Characteristics of the Physicians	n (%)
**Gender**	
Men	182 (56%)
Women	143 (44%)
**Clinical specialty**	
General practice	156 (48%)
Internal medicine	104 (32%)
Obstetrics and gynecology	65 (20%)
**Average weekly time spent gaining medical knowledge**	
Less than 1 hour	65 (20%)
1-2 hours	182 (56%)
3-5 hours	78 (24%)
**Source of medical knowledge preferred**	
Internet	169 (52%)
Medical books	117 (36%)
CMEs	39 (12%)

The responses of physicians to various questions regarding their perspective of the increasing trend of direct to consumer marketing of nutraceuticals is shown in Table-II. It showed that most physicians experience knowledgeable patients who inquire about their diseases and the treatment plan, however, very few physicians believed that either their knowledge is enough to deal with those patients or the information provided by the pharmaceuticals. However, most of the physicians believed that e-detailing and DTCA of nutraceuticals helps practitioners in shaping a more effective treatment plan.

**Table-II:** Physicians’ perspective regarding Direct to Consumer advertisement of Nutraceuticals.

Physicians’ Perspective regarding direct to consumer advertisement of nutraceuticals	n (%)
Patients inquiring questions regarding their disease and treatment	286 (88%)
Physician knowledge is sufficient and updated enough to handle the questions of patients	26 (8%)
Patients will opt for other physicians if they do not satisfactorily answer their queries	247 (76%)
Knowledge provided by pharmaceuticals is enough for HCP to choose drugs to prescribe	91 (28%)
Social media helps HCP understand patients’ evolving health seeking behavior	208 (64%)
DTC marketing of nutraceuticals profoundly impacts patients’ choices	104 (32%)
DTC marketing of nutraceuticals helps practitioners in shaping a more effective treatment plan	221 (68%)
e-detailing of nutraceuticals helps practitioners in shaping a more effective treatment plan	234 (72%)
HCP prescribe medications on patients request	201 (62%)

DTC: Direct to consumer

## DISCUSSION

Most of the participants in our study were men, who were general practitioners, utilized internet to update their medical knowledge and spent one to two hours for this purpose. Very few HCPs believe that their knowledge is wholesome enough to satisfy the queries of their patients. Only one-fourth of the HCPs find the information provided by pharmaceuticals to be sufficient enough to deal with patient queries. Almost three-fourth of the participants perceived e-detailing and DTCA of nutraceuticals to play a significant role in determining the treatment plans. Hence, most HCPs take help from social media to understand the evolving behavior of their patients. Only one-third participants felt that DTCA has a profound influence on the prescription priorities of their patients. Almost two-third HCPs believe that DTCA will help them in shaping a more effective treatment plan.

While research into the effects of e-detailing and e-marketing of nutraceuticals on consumer behavior is progressing substantially^14^, the physicians’ perspective has been a relatively lesser studied domain, especially in the local set up. Previously, DTCA and e-marketing was regarded as a facilitative means to open communication opportunities for the patients, it turned out to be the force destabilizing the physician’s role as the authoritative and trusted source of medical knowledge. The quantitative evidence available in medical literature suggests high uncertainty and fluctuation in the effects of e-marketing and DTCA of nutraceuticals.

When patients are loaded with medical information through DTCA, some physicians find it time-consuming to deal with them, and end up submitting to their request of prescription as long as patient health is not compromised.^15^

As consistent with our study, other researchers have also agreed to a significant influence of DTCA on the physician-patient relationship by compelling patients to request specific prescriptions and also elevating the expectations from the physician’s prescribing practices. Consequently, the physicians might deviate from standard prescribing practice in order to achieve patient satisfaction by abiding to their demands.^16^,^17^

This implies that the role of DTCA and e-marketing in nutraceuticals is still ambivalent. Where it has its benefits, the shortcomings can also not be neglected. Hence, with the trend of DTCA gaining popularity, the responsibility of appropriate prescription, and proper patient counselling regarding drug choices and their effects, increases at the physicians’ end.

## CONCLUSION

Physician perception of DTCA of nutraceuticals seems to be promising. Digital marketing has a proven role in consumer behavior, hence pharmaceuticals also need to prioritize it as a publicity medium. Physicians understand the impacts of DTCA on the behavior of their patients. Physicians must be fully prepared to deal with knowledgeable patients and put in maximum efforts to counsel them in such a manner that the prescription doesn’t contain “request specific” drugs but only the ones that are most beneficial for the patients.
